# Five Calculi Removed by Lithotomy, Each Containing a Field Bean as a Nucleus

**Published:** 1852-06

**Authors:** 


*Five Calculi Removed by Lithotomy, each containg a Field Bean as a Nucleus.*—The following remarkable case is related by Dr. Mackenzie:—A laborer, aged 46, was admitted into the Edinburgh Infirmary with the usual symptoms of stone. On sounding, the presence of more than one calculus was ascertained. The lateral operation was performed on the 13th of October, and five stones were removed. The prismatic shape and uniform size of these were remarkable; but the presence of a foreign body as a nucleus was not suspected until the stones had been dried by evaporation, when a hard substance was heard to rattle loosely within them. On making sections of these calculi, the nuclei were found to be horse-beans. The calculous incrustations consisted of the triple phosphates.

The history of this remarkable case is as follows:—About the end of March of the present year, after a carousal with two fellow-laborers, with whom he lodged in a barn attached to his master's farm, a quarrel arose, in which he was knocked down and overpowered by his two companions. From the injuries he received, and from his state of intoxication, he was rendered senseless, and, whilst in this condition, the following cruel trick was perpetrated on him by his assailants:—

He was stripped of his clothes, and a quantity of beans

(the common field or horse-beans, used for feeding cattle) were thrust into his mouth and into the rectum; and lastly, several were introduced into his urethra. The manner in which these found their way into the bladder is unknown, but it is probable that several were introduced, one after another, into the orifice of the urethra, and then pushed back along the canal by the pressure of the fingers on the penis and perineum.

On the following morning he was found in a state of insensibility, with his genital organs covered with blood. His companions had made off, and have ever since escaped detection.

A number of beans were vomited, and passed *per anum* on the day following the assault, and during this and the subsequent day he suffered great pain in voiding his urine, which was mixed with blood, and contained several fragments of broken beans.

He was confined to bed for some days, but at the end of a week he had nearly recovered from his injuries, and his urinary symptoms had considerably abated in severity.

From that time forward, however, he continued to suffer more or less severely from the usual symptoms of stone in the bladder, which were well marked at the time of his admission into the hospital.—*Edinburgh Monthly Journal, Jan.* 3, 1852.

